# Oxygen Imaging for Non-Invasive Metastasis Detection

**DOI:** 10.3390/s22010237

**Published:** 2021-12-29

**Authors:** Joshua Punnoose, Henry Nachman, Shai Ashkenazi

**Affiliations:** 1 Department of Biomedical Engineering, University of Minnesota, Minneapolis, MN 55455, USA; punno002@umn.edu; 2 Department of Physics and Astronomy, University of North Carolina, Chapel Hill, NC 27599, USA; henry.nachman@unc.edu

**Keywords:** head and neck cancer, imaging, oxygen imaging, photoacoustic, sentinel lymph node biopsy

## Abstract

Sentinel lymph node (SLN) biopsy is an integral part of treatment planning for a variety of cancers as it evaluates whether a tumor has metastasized, an event that significantly reduces survival probability. However, this invasive procedure is associated with patient morbidity, and misses small metastatic deposits, resulting in the removal of additional nodes for tumors with high metastatic probability despite a negative SLN biopsy. To prevent this over-treatment and its associated morbidities for patients that were truly negative, we propose a tissue oxygen imaging method called Photoacoustic Lifetime Imaging (PALI) as an alternative or supplementary tool for SLN biopsy. As the hyper-metabolic state of cancer cells significantly depresses tissue oxygenation compared to normal tissue even for small metastatic deposits, we hypothesize that PALI can sensitively and specifically detect metastases. Before this hypothesis is tested, however, PALI’s maximum imaging depth must be evaluated to determine the cancer types for which it is best suited. To evaluate imaging depth, we developed and simulated a phantom composed of tubing in a tissue-mimicking, optically scattering liquid. Our simulation and experimental results both show that PALI’s maximum imaging depth is 16 mm. As most lymph nodes are deeper than 16 mm, ways to improve imaging depth, such as directly delivering light to the node using penetrating optical fibers, must be explored.

## 1. Introduction

Accurate assessment of tumor spread is essential for determining patient prognosis and treatment planning. This is particularly true for head and neck cancer patients where the presence of metastasis reduces 5 year survival by ~50% [1]. Initial evaluation of lymph node metastases is done by palpating the neck to identify enlarged nodes. However, this method is operator dependent and has a low sensitivity of 67% [2]. This low sensitivity places patients at risk for distant tumor recurrence which is associated with a 5-year survival rate of 35% [3]. To better identify patients with metastasis, palpation is supplemented with morphological and/or functional imaging. In morphological imaging, metastatic nodes are identified by analyzing physical markers of metastasis, such as node size, shape, and the presence of a necrotic core [4]. However, morphological characteristics are poor predictors of metastasis. When imaging necks initially diagnosed as metastasis-free, one meta-analysis found that CT, MRI, and US had a sensitivity of 52%, 65%, and 66%, respectively [5]. Functional imaging can detect molecular changes in the tissue and is thus a promising modality for improving the sensitivity of metastasis detection. However, PET, a functional imaging modality commonly used for metastasis detection in head and neck cancer, achieved a sensitivity of 66% due to its inadequate image resolution [5]. The lack of sensitivity in both palpation and imaging leads some clinicians to remove all lymph nodes in the tumor region for patients with aggressive cancer phenotypes despite negative palpation and imaging results. As metastasis occurs in 20–30% of cases, this treatment strategy overtreats 70–80% of patients [2] and unnecessarily puts them at risk for the morbidities associated with surgery, such as lymphedema and reduction in shoulder functionality.

More accurate diagnosis can be achieved with sentinel lymph node (SLN) biopsy which involves excising the nodes directly connected to the tumor and sending them to a lab for immunohistochemical staining. However, this process cannot be done intraoperatively, thus requiring positive biopsy patients to return to the hospital for a second surgery to treat the metastasis [6]. Additionally, a prospective study of the effects of SLN biopsy for 29 early stage head and neck cancer patients found that 17% of SLN biopsy patients had lymphedema [7]. 

To improve the sensitivity and specificity of nodal assessment and prevent over-treatment, we propose a new functional imaging modality called photoacoustic lifetime imaging (PALI) [8,9]. PALI is an optical, non-invasive technique that provides tissue oxygen mapping and is thus sensitive to cellular metabolism. As numerous works have demonstrated reduced tissue oxygenation in metastatic nodes compared to normal nodes [10,11,12,13,14], we hypothesize that PALI’s tissue oxygen imaging capabilities can be used to sensitively and specifically detect metastatic deposits. Further evidence of oxygen as a valuable identifier of metastasis was shown by Luke et al. who demonstrated detection of metastases volumes as small as 2.6 × 10^−3^ mm^3^ using blood oxygenation measurements in a metastatic mouse model [15]. As tissue oxygen measurements are more sensitive to oxygenation changes than blood oxygenation measurements, PALI may be able to detect similar volumes at a higher sensitivity and specificity than reported by Luke et al. (sensitivity and specificity of 71% and 83%, respectively) [15]. Additionally, as a non-invasive lymph node monitoring technique, PALI would enable longitudinal assessment of nodal status to ensure continued locoregional control following treatment.

Towards this goal, PALI’s imaging depth must first be evaluated to identify whether the optics underlying PALI can penetrate the highly attenuating tissue medium to depths relevant to cancers that use SLN biopsy. In this report, PALI’s applicability for head and neck cancers is evaluated using both simulations and benchtop experiments. While the scope of the paper was narrowed to head and neck cancers, this technique could be extended to treat other cancers where SLN biopsy is done [16,17,18,19,20].

## 2. Materials and Methods

### 2.1. Photoacoustic Lifetime Imaging

**Figure 1:**
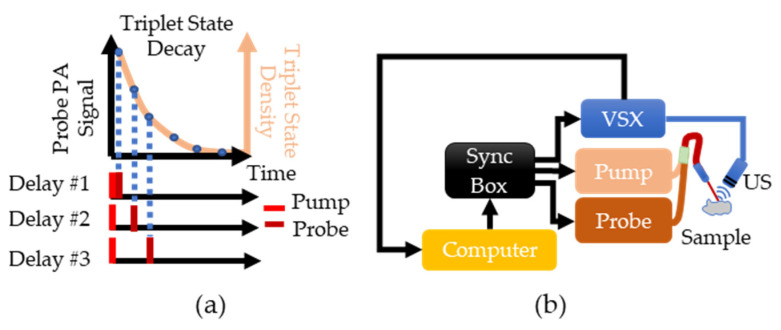
(**a**) Overview of PALI theory. The beige line shows the expected exponential decay of the triplet state. This decay is sampled by varying the delay between the pump and probe lasers and measuring the photoacoustic signal generated by the probe. (**b**) Implementation of PALI begins with the computer where a MATLAB script is used to program a delay into the synchronization box. This box then triggers the pump, the probe, and the Verasonics (VSX) system. Triggering VSX initiates data acquisition by the ultrasound (US) probe. The recorded signal is then transferred to the computer for further processing.

PALI measures pO_2_ using a pump-probe approach where one laser “pumps” methylene blue (MB) molecules to an excited state called the triplet state, and another laser “probes” the density of molecules in this state as it decays to the ground state. The decay rate of the triplet state is measured by varying the delay between the pump and the probe to sample the triplet state density at different time points along its decay, as shown in Figure 1a. The absorption of the probe laser pulse by the triplet state generates a photoacoustic wave whose magnitude is proportional to the triplet state density. The triplet state decay rate is found by fitting the measured photoacoustic signals to a decaying exponential. By assuming that triplet MB mainly reacts with oxygen, the Stern-Volmer equation can be used to relate the triplet state decay rate with oxygen concentration: $k_{Q}\left[O_{2}\right]+k_{0}=k_{t}$. In this equation, $k_{Q}$ is the reaction rate between oxygen and triplet state MB, $k_{0}$ is the oxygen-independent decay rate, [$O_{2}]$ is the oxygen partial pressure, and $k_{t}$ is the triplet state decay rate. By doing a calibration where [$O_{2}]$ is varied and the corresponding $k_{t}$ is measured, $k_{Q}$ and $k_{0}$ can be calculated and enable the conversion of future decay rate measurements into an oxygen partial pressure.

To implement PALI, two tunable, pulsed lasers (Phocus, Opotek, Carlsbad, CA, USA) connected via a bifurcated fiber bundle and a research ultrasound system called Verasonics (VSX) (Vantage 64 LE, Verasonics, Kirkland, WA, USA) are triggered by a synchronization box (Intel Max 10, Intel, Santa Clara, CA, USA) to control the timing of the pump pulse, probe pulse, and signal acquisition, as shown in Figure 1b. A single element ultrasound transducer (V309-SU, Olympus, Waltham, MA, USA) is used to sense the photoacoustic waves and the signal is then stored within VSX. The pump laser was tuned to 660 nm and the probe laser was tuned to 830 nm as the ground and triplet state have a maximum absorption at these wavelengths [21].

Although only the photoacoustic signals generated by the probe laser are used for data analysis, both the pump and probe lasers generate a signal. The pump generates a signal due to ground state MB absorption while the probe signal is from the triplet state absorption. As a result, the probe photoacoustic signal is corrupted by the pump. To remove this artifact, for each delay, the pulsing sequence shown at the bottom of Figure 1a is repeated except the probe laser is not fired. The resulting signal is subtracted from the signal measured when both the pump and the probe are pulsed. For each delay, 100 pumps and probe and 100 pump only photoacoustic signals were collected and averaged to improve the signal-to-noise ratio (SNR). All signals were normalized by pulse energy, and twelve delays evenly spaced between 0.5 and 1000 µs on the logarithmic scale were used to ensure even sampling of all regions of the decay. To remove the background signal originating from non-MB absorption, the PA signal at a delay corresponding to the complete decay of the triplet state is subtracted from all other delays.

### 2.2. PALI Simulation

**Figure 2:**
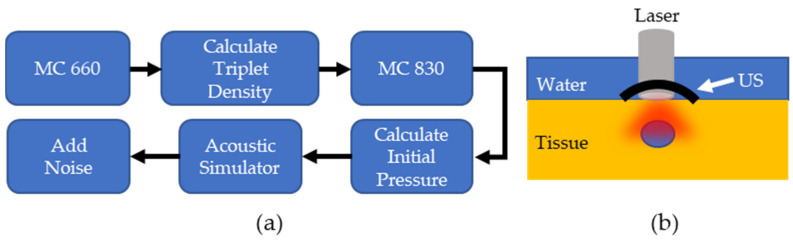
(**a**) Overview of PALI Simulation. MC: Monte Carlo simulation (**b**). Computational phantom to measure PALI imaging depth.

The PALI simulation consisted of two parts: a Monte Carlo and an acoustic wave simulation. An overview of the simulation workflow for each lymph node depth and the computational phantom setup is shown in Figure 2a,b, respectively. For the Monte Carlo simulation, the MCmatlab software package was used to design a phantom with the same optical properties as neck tissue at the pump and probe wavelengths [22]. The tissue was set to a µ_a_ of 0.22 and 0.235 $\frac{1}{cm}$ and $\mu_{s}^{\prime }$ was set to 9.1 and 7.3 $\frac{1}{cm}$ for 660 nm and 830 nm, respectively. Within the optical phantom, a spherical lymph node was created containing 400 µM MB. In solution, MB exists in equilibrium between a monomer and dimer state, where dimerization is favored at higher concentrations. While both species exhibit oxygen dependent decay, PALI aims to measure the monomer decay rate as only monomers decay at a rate that falls within the measurement capabilities of our system for the full range of clinically relevant oxygenation values. 400 µM was chosen as this is the maximal concentration where the photoacoustic signal from the monomers will be greater than that of the dimers.

The light source used for this simulation was a circular beam perpendicular to the surface of the tissue at an energy of 10 $\frac{mJ}{cm^{2}}$ for the pump and 15 $\frac{mJ}{cm^{2}}$ for the probe, which matches the pulse energies of the lasers used in the experiment. The pump fluence distribution outputted from MCmatlab is used to calculate the triplet population within the lymph node using Equation (1), whose derivation is relegated to Appendix A.

(1) $$n_{t}=\frac{\beta n}{\alpha +\beta }\left(1-e^{-\left(\alpha +\beta \right)t}\right)$$

In Equation (1), $n_{t}$ is the concentration of triplet state molecules, β is the rate of molecules entering the triplet state, n is the total concentration, α is the triplet state decay rate, and t is the pulse duration. $\beta =\frac{\eta \sigma_{g}\phi_{660}}{h\nu }$ where η is the triplet quantum yield for MB, $\sigma_{g}$ is MB’s ground state absorption cross-section, $\phi_{660}$ is the fluence rate in units of $\frac{W}{cm^{2}}$, h is Planck’s constant, and ν is the frequency of the light.

The calculated triplet concentration is then multiplied by its molar extinction coefficient [21] to calculate the triplet state’s absorption coefficient, and this is used to update the absorption coefficient in the methylene blue filled lymph node. A second Monte Carlo simulation determines the fluence distribution at the probe wavelength. This is used to calculate the probe photoacoustic signal according to Equation (2), where Γ is the Grüneisen parameter, a unitless parameter describing the conversion efficiency of heat into a photoacoustic signal, $\mu_{a}$ is the absorption coefficient of triplet state methylene blue, and $\phi^{830}$ is the fluence in units of $\frac{J}{cm^{2}}$.

(2) $$P=\Gamma \mu_{a}\phi^{830}$$

**Figure 3:**
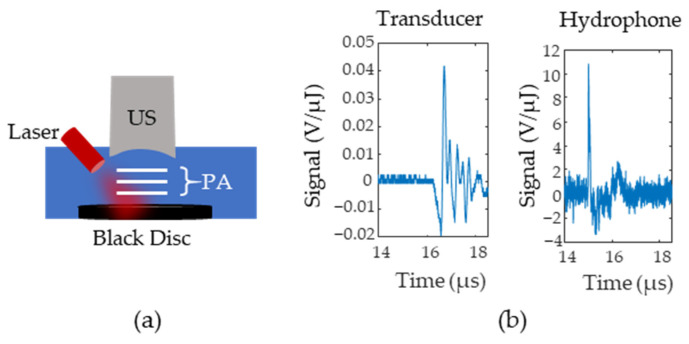
(**a**) Setup for Noise Equivalent Pressure (NEP) measurements. A black disc is illuminated and the photoacoustic signals (P(A) are first measured with an ultrasound (US) transducer and then replaced with a hydrophone (H). (**b**) Example signals from the transducer and the hydrophone. Signals were normalized according to the laser pulse energy.

The initial pressure distribution from the probe is fed into an acoustic simulator called K-Wave [23], a software that allows users to define the medium’s acoustic properties, the acoustic source geometry, and the acoustic sensor geometry. The speed of sound of the medium was set to 1540 $\frac{m}{s}$ and the tissue was assumed to have negligible acoustic attenuation. A 5 MHz, single element, focused ultrasound transducer was used as the acoustic sensor and was placed directly above the lymph node. To obtain the radiofrequency (RF) data for different time delays, the data was multiplied by a decaying exponential with a rate that corresponded to the pre-defined oxygenation level of the lymph node. The lymph node oxygenation was fixed to either 150 mm Hg or 0 mm Hg. This process was repeated for lymph node depths from 4 to 25 mm in steps of 3 mm. The amount of additive noise included in the simulation was approximated using the setup in Figure 3a where a black disc was 3D printed and placed at the bottom of a water tank at the focal point of a single element transducer. The photoacoustic signal generated by the disc was measured with both a hydrophone and a single element transducer. Figure 3b shows the transducer (left side) and hydrophone (right side) responses after being normalized by pulse energy (measured in µJ). Equation (3) calculates the noise equivalent pressure (NEP) and defines the standard deviation of a zero mean Gaussian noise distribution. In Equation (3), $U_{noise}$ is the root mean square of the signal for samples before the arrival of the photoacoustic wave, $H_{sig}$ is the peak-to-peak value of the hydrophone response to the photoacoustic wave, and $U_{sig}$ is the peak-to-peak value of the ultrasound transducer’s response.

(3) $$NEP=U_{noise}*\frac{H_{sig}}{U_{sig}}$$

### 2.3. Experimental Setup

**Figure 4:**
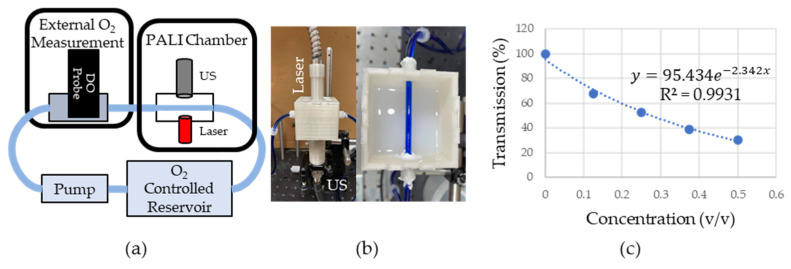
(**a**) Overview of phantom setup. Fluid from O_2_ controlled reservoir was pumped into a box where oxygen measurements were collected with PALI using an ultrasound (US) transducer and laser. These measurements were verified using a dissolved oxygen probe (DO). (**b**) Picture of PALI chamber. Phantom consisted of 3D printed box with holders for the laser and ultrasound transducer. Tubing containing 400 µM MB was placed at the focal point of the transducer. (**c**) Measurement of the effective attenuation coefficient of scattering fluid. Dots represent the measured transmission of 660 nm light through the medium. The dashed line represents the exponential fit whose rate is in units of $\frac{1}{cm*f}$ where f is the fractional volume of the scattering fluid relative to the final fluid volume. Since the fluid was not diluted further (i.e., the fractional volume is 1) the effective attenuation is 2.34 $\frac{1}{cm}$.

An overview of the phantom system is shown in Figure 4a. Briefly, a peristaltic pump (AE1207, Gikfun, Dongguan, Guangdong, China) pulled 400 µM MB from a room-temperature, oxygen-controlled reservoir into the dissolved oxygen (DO) chamber where a DO probe (ENV-40-DOX, Atlas Scientific, New York City, NY, USA) provided a reference oxygen measurement. The oxygen in the MB reservoir was controlled by bubbling either room air (for a high oxygen solution) or argon (for a low oxygen solution). The solution then entered the PALI measurement chamber shown in Figure 4b which consisted of Tygon (Saint-Gobain, Malvern, PA, USA) tubing running through the center of a 3D printed box with a 5 MHz single element transducer located on the box’s bottom and a laser fiber bundle at the top. The tubing was placed at the focal point of the transducer for maximum sensitivity and a single element transducer geometry was chosen due to its higher sensitivity compared to array transducers. The laser was placed perpendicular to the tube to minimize specular reflection at the air-phantom interface. The box was then filled with an Intralipid and India Ink mixture, shown on the right side of Figure 4b, that approximated the optical properties of neck tissue. To design this mixture, first, the optical properties of the Intralipid and the India Ink alone were measured. It was assumed that the absorption in the Intralipid and scattering in the India Ink was negligible compared to their scattering and absorption, respectively. To measure the dependence of scattering and absorption on Intralipid and India ink concentration, respectively, varying dilutions of these solutions were placed between a 660 nm laser source (Civil Laser, Hangzhou, Zhejiang, China) and a photodetector (Det10A, ThorLabs, Newton, NJ, USA). The change in transmittance was measured and fitted to a decaying single exponential. Since the distance, the light traveled through the mixture was fixed and the concentration was known, a constant could be calculated that related the scattering or absorption coefficient to the concentration. This was then used to calculate the concentration necessary to achieve the desired optical properties. Figure 4c shows the percent transmission in the Intralipid and India Ink solution as the fractional concentration of the optical fluid is varied. In the plot, 100% transmission corresponds to the transmission through water. Transmission measurements are shown by the blue round dots. The data was fit to a model of light transport in diffusive media ($e^{-\mu_{eff}d}$) where $\mu_{eff}$ is the effective attenuation coefficient and d is the distance the light travels through the medium. After fixing d to 1 cm, which is the distance the light travels through the cuvette, $\mu_{eff}$ was found by fitting the data. $\mu_{eff}$ was found to be 2.34 $\frac{1}{cm}$, close to the desired effective attenuation of 2.5 $\frac{1}{cm}$. The fit is shown as a dashed blue line in Figure 4c.

To simulate acquiring measurements at varying tissue depths, the height of the Intralipid/India ink mixture above the tubing was varied from 1 mm to 16 mm in steps of 3 mm and each measurement was repeated seven times. Measurements were collected for high and low oxygen solutions. The ground truth PALI measurement was found by replacing the scattering fluid with water and acquiring seven measurements. For each depth, the mean, standard deviation, and percent error from the ground truth were calculated. The clinical imaging depth was defined as the depth where the average PALI measurement had an error greater than 10 mm Hg. 10 mm Hg was chosen as the error threshold as it provided 95% confidence in classifying 90% of metastatic nodes as not normal and 95% of normal nodes as not metastatic based on the data provided by Becker et al. [10]. The measurement depth limit was defined as the depth where 0% error was beyond two standard deviations from the average error.

**Figure 5:**
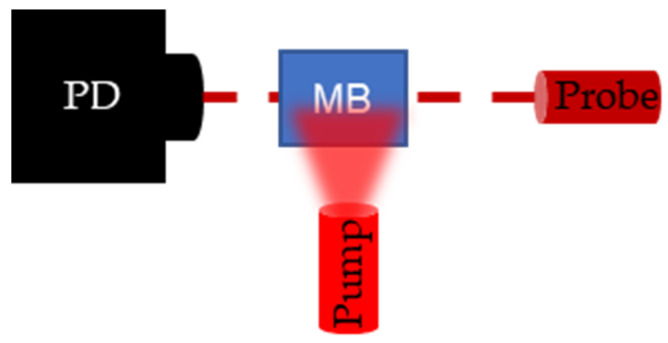
Setup for flash photolysis experiment. Photodetector (PD) measures the change in transmission of the continuous wave probe laser following a pulse excitation by the pump laser. When the pump excites the methylene blue (M(B), the transmission of the probe laser decreases and transmission recovers as the triplet state density goes to 0.

To validate PALI-derived decay rates, flash photolysis measurements were collected. Flash photolysis involves measuring the change in transmission of a continuous wave probe laser as ground state MB molecules are pumped to the triplet state. A top view of the flash photolysis setup is shown in Figure 5. A photodetector is placed (PD) (Det10A, ThorLabs, Newton, NJ, USA) opposite a laser diode (L840P200, ThorLabs, Newton, NJ, USA) set to the probe wavelength, and a fiber bundle, delivering the pump wavelength, is placed perpendicular to the probe trajectory. The dark red dashed line denotes the trajectory of probe light through the sample and the light red, blurred triangle represents the broad beam pump illumination. Pumping the MB molecules to the triplet state results in a drop in the transmission percentage which recovers over time as triplet molecules decay to the ground state. By connecting the photodetector to the oscilloscope, the decay can be recorded and the decay rate measured.

## 3. Results and Discussion

**Figure 6:**
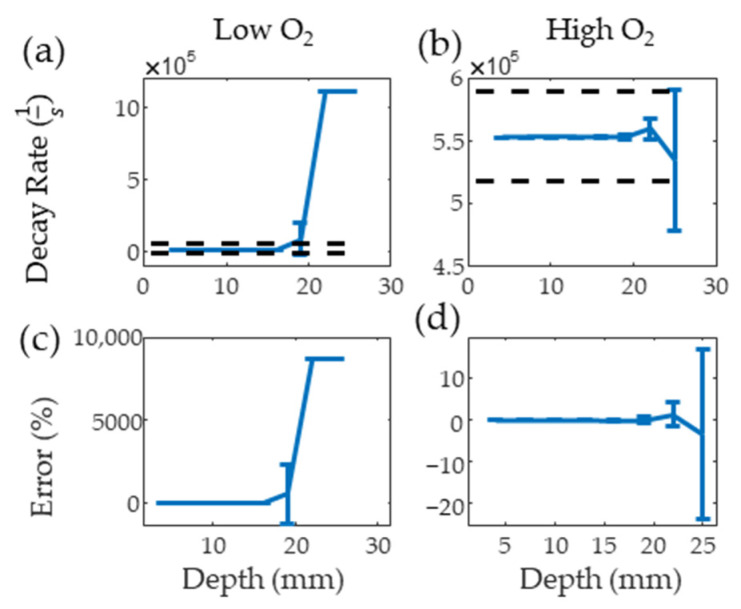
Mean and standard deviation of simulated PALI decay rate measurements at varying depths for deoxygenated (**a**) and oxygenated (**b**) solutions. Black dashed lines represents the decay rate corresponding to 10 mm Hg error. Error from the true decay rate for low and high oxygenations is shown in (**c**,**d**).

Figure 6a,b show the results from the PALI simulation at varying depths for low and high oxygen solutions along with black dashed lines indicating the decay rate that results in 10 mm Hg error. The average decay rate with one standard deviation error bar is shown for each depth. The clinical imaging depth for low and high oxygen solutions is below 19 and beyond 25 mm, respectively. Figure 6c,d show that the measurement depth limit is below 22 and beyond 25 mm for deoxygenated and oxygenated solutions, respectively. As the average lymph node depth for head and neck cancer is 25 mm [24], methods of improving signal from the node, such as delivering light directly to the node using optical fibers, increasing nodal MB concentration, or increasing surface light energy, must be explored for this technology to be translated to the clinic.

**Figure 7:**
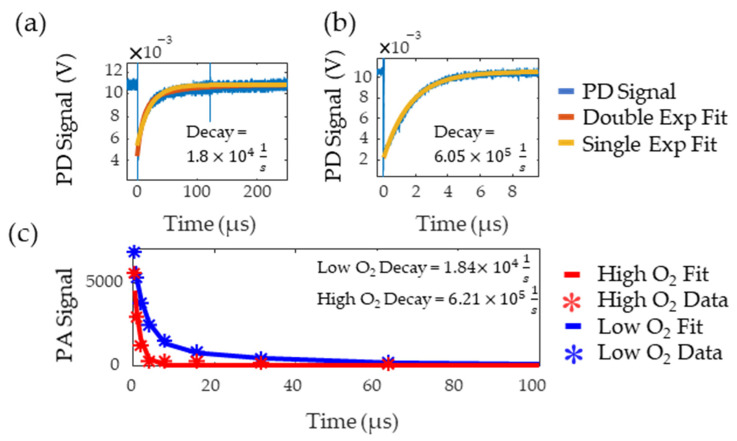
Low (**a**) and high (**b**) oxygen decay rate measured by flash photolysis. The blue line is the photodetector (PD) signal, red is the double exponential fit, and yellow is the single exponential fit. (**c**) Decay rate measured by PALI for tubing in water. Stars are the data points and the lines are the fit. The red and blue lines correspond to high and low oxygen, respectively.

Figure 7a,b show the flash photolysis signal collected for high and low oxygen levels, respectively, along with single and double exponential fits. Based on previous works [8,9], the data was expected to follow a single exponential but, for the low oxygen MB solution, this resulted in poor goodness of fit as it failed to capture the initial part of the decay. A double exponential better fit this data, suggesting the presence of additional reactants with the triplet state. The slower decay rate from the double exponential was recorded as this rate consistently matched the literature values for triplet state lifetime in low oxygen solutions [21]. The decay rates of the fit were $6.05\times 10^{5}$ and $1.84\times 10^{4}$ $\frac{1}{s}$, for the oxygenated and deoxygenated solutions, respectively. Figure 7c shows the decay rate measured with PALI for tubing in water. PALI accurately captures the double and single exponential behavior observed in the flash photolysis results for the low and high oxygen MB solutions, respectively, and reports a decay rate that is consistent with that measured by flash photolysis. Fitting was done using the Levenberg-Marquardt algorithm and the model that was fit to the deoxygenated data is shown in Equation (4). The scale factor for the second exponential was set to (1−a) as it was assumed that only two decay rates were present in the solution.

(4) $$ae^{-bt}+\left(1-a\right)e^{-c*t}$$

**Figure 8:**
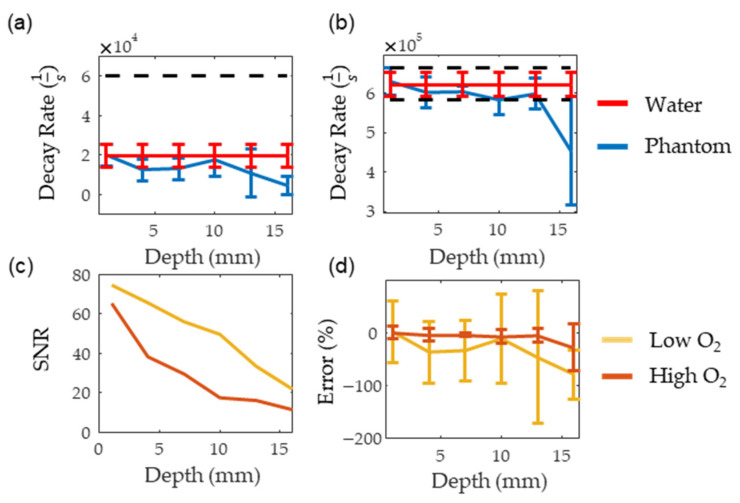
(**a**,**b**) show the measured decay rate at different depths for deoxygenated and oxygenated solutions, respectively. The red line represents the decay rate measured in water while the blue line is the decay rate measured in the scattering medium. (**c**,**d**) show the SNR and the error for the oxygenated (dark red) and deoxygenated (dark yellow) solutions, respectively.

Figure 8a,b show the consistency of PALI measurements at different depths along with a black dashed line indicating the decay rate corresponding to the 10 mm Hg threshold. The decay rate associated with 10 mm Hg was calculated using a two-point calibration with PALI measurements at oxygenated and deoxygenated solutions. The depth where the decay rate error exceeded 10 mm Hg was beyond 16 mm for the deoxygenated solutions and at 16 mm for oxygenated solutions. As the standard deviation of the deoxygenated solution is expected to be similar to the standard deviation of metastatic node measurements, this data indicates that PALI may achieve high sensitivities in identifying metastatic nodes for depths beyond 16 mm in the tissue. However, at 16 mm PALI measurements at low oxygenations become unreliable as shown in Figure 8d where the mean and two standard deviation error bars are shown. As 0% error is beyond two standard deviations from the mean, less than 2.5% of collected measurements are expected to be near the actual oxygenation. Figure 8c shows that this depth corresponds to an SNR of 20. In contrast, for high oxygen solutions, PALI can achieve less than 10 mm Hg error for depths up to 16 mm with a corresponding SNR of 10 but reliable measurements can be achieved for depths beyond 16 mm. Simulation, however, predicts that accurate and reliable measurements can be measured below 22 mm and beyond 25 mm for deoxygenated and oxygenated solutions, respectively. A potential source for this discrepancy may be differences in MB monomer concentration. Using kinetic equations, it was calculated that at 400 µM, about 50% of the MB concentration would be monomers. However, the actual concentration of monomer MB available for reaction with oxygen may be less than this value due to the presence of other reactions with MB monomers or to dimerization.

As both the illumination and photoacoustic sensing geometry were optimized in this setup, this depth represents the maximum PALI imaging depth. As lymph node depths for many types of cancers are beyond this [24,25], methods to increase the SNR must be explored. One potential method would be to use penetrating optical fibers to illuminate the lymph node rather than using trans-dermal illumination. Further work must be done to assess the fraction of the lymph node’s volume that can be illuminated with this method and how to optimize energy coupled into the fiber. Another option would be to increase the concentration of monomer MB within the lymph node by embedding it in a complex, such as cucurbituril [26]. While increasing the concentration does increase SNR, it limits the penetration depth of light within the lymph node due to the high absorption coefficient of MB. This in turn limits oxygen mapping to only superficial regions of the node. More work must be done to identify the optimal MB concentration for node imaging. While a single element transducer was used in this study due to its high ultrasound sensitivity, array transducers are more useful in the clinical setting as they provide faster imaging. One geometry that is particularly interesting are concave ring arrays [27] as it enables high ultrasound sensitivity, illumination perpendicular to the skin, and fast imaging. Reanalyzing PALI imaging depth with a more clinically relevant transducer geometry is another future step for this project.

## 4. Conclusions

Sentinel lymph node biopsy is an essential tool to understand the patient prognosis and determine treatments. However, current lymph node assessment methods are either invasive and cause pain or lack adequate sensitivity or specificity. By measuring tissue oxygen, PALI may better identify metastatic lymph nodes compared to current technologies due to the hyper-metabolic state of cancer cells compared to normal tissue. Simulation and benchtop experiments were done to assess the maximum depth that PALI can obtain measurements with less than 10 mm Hg error and the depth where PALI provides reliable decay rates for oxygenated and deoxygenated solutions. Experimentally, it was found that below 16 mm, PALI can reliably measure the decay rate of oxygenated and deoxygenated solutions and achieve a mean error of less than 10 mm Hg. Simulation results predict a higher depth limit of 22 mm. This discrepancy may be due to the presence of MB dimers and work must be done to prevent aggregation to improve imaging depth. Since the sentinel lymph node for many cancers is often deeper than 16 mm, implementing PALI with penetrating optical fibers must be explored. Specifically, the question of how many fibers and their geometry around the node must be studied. While the preliminary results from this study are encouraging, more work must be done to evaluate the system’s sensitivity and specificity in identifying metastases using in-vivo models. These models will account for the non-uniformity of dye concentration within the lymph node, the presence of additional reactions with triplet MB, and the acoustic attenuation of tissue.

## Figures and Tables

**Figure A1:**
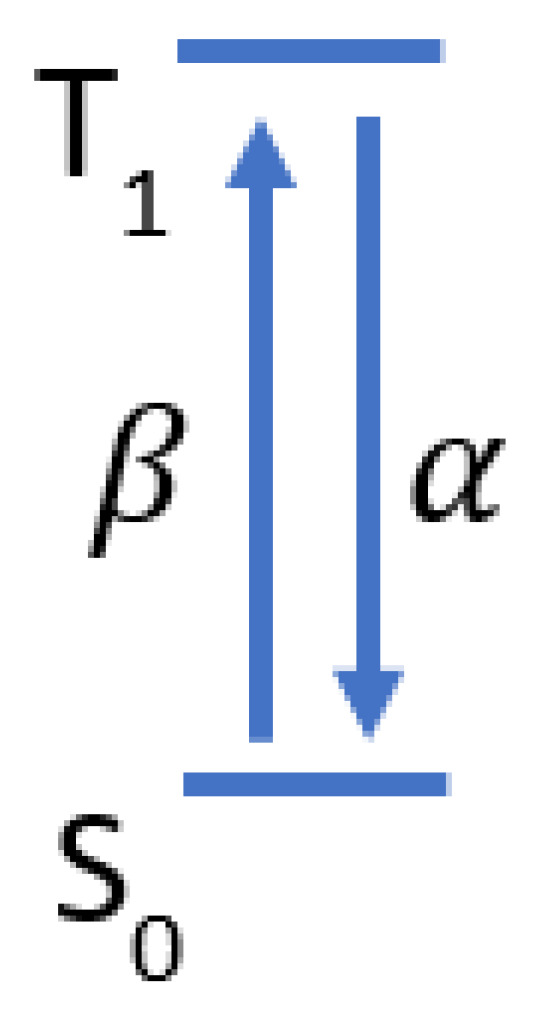
Two state model of methylene blue triplet state dynamics.

## Data Availability

Publicly available datasets were analyzed in this study. Code used to anlayze the data can be downloaded here: https://github.umn.edu/punno002/PALI-Imaging-Depth-Lymph-Node (accessed on 27 December 2021). Due to its large size, the data could not be posted on GitHub but will be made available upon request. Send all data requests to aske003@umn.edu.

## Appendix A

A two-state model of the methylene blue dynamics is shown in Figure A1 where $S_{0}$ is the ground state and $T_{1}$ is the triplet state. The transition rate from $S_{0}$ to $T_{1}$ is β and decay rate from $T_{1}$ to $S_{0}$ is α. Given this, the change of the number of molecules in $T_{1}$ can be modeled using Equation (A1) where $n_{t}$ is the number of molecules in the triplet state and $n_{0}\;$is the number of molecules in the ground state.

(A1)
$$\frac{dn_{t}}{dt}=\beta n_{0}-\alpha n_{t}$$

(A2)
$$n=n_{0}+n_{t}$$

Equation (A2) reflects that the only place molecules can go in our system is the triplet or ground state. Equation (A3) shows the solution for triplet state dynamics in terms of $n_{T}$ with the initial condition that $n_{t}\left(t=0\right)=0$.

(A3) $$n_{t}=\frac{\beta n}{\alpha +\beta }\left(1-e^{-\left(\alpha +\beta \right)t}\right)$$

β is dependent on the number of ground state molecules that absorb the excitation light as shown in Equation (A4) where η describes the percentage of ground state molecules that absorbed the excitation light that enter the triplet state, I describes the intensity of the excitation beam in units of $\frac{W}{cm^{2}}$, $\sigma_{0}$ is the ground state absorption cross-section in units of $\frac{1}{cm^{2}}$, h is Planck’s constant, and ν is the frequency of the excitation beam.

(A4) $$\beta =\frac{\eta I\sigma_{0}}{h\nu }$$
